# The Role of Methionine Residues in the Regulation of Liquid-Liquid Phase Separation

**DOI:** 10.3390/biom11081248

**Published:** 2021-08-21

**Authors:** Juan Carlos Aledo

**Affiliations:** Departamento de Biología Molecular y Bioquímica, Facultad de Ciencias, Universidad de Málaga, 29071 Málaga, Spain; caledo@uma.es

**Keywords:** methionine sulfoxide, biomolecular condensate, stress granule, ataxin-2, TDP43, Pab1

## Abstract

Membraneless organelles are non-stoichiometric supramolecular structures in the micron scale. These structures can be quickly assembled/disassembled in a regulated fashion in response to specific stimuli. Membraneless organelles contribute to the spatiotemporal compartmentalization of the cell, and they are involved in diverse cellular processes often, but not exclusively, related to RNA metabolism. Liquid-liquid phase separation, a reversible event involving demixing into two distinct liquid phases, provides a physical framework to gain insights concerning the molecular forces underlying the process and how they can be tuned according to the cellular needs. Proteins able to undergo phase separation usually present a modular architecture, which favors a multivalency-driven demixing. We discuss the role of low complexity regions in establishing networks of intra- and intermolecular interactions that collectively control the phase regime. Post-translational modifications of the residues present in these domains provide a convenient strategy to reshape the residue–residue interaction networks that determine the dynamics of phase separation. Focus will be placed on those proteins with low complexity domains exhibiting a biased composition towards the amino acid methionine and the prominent role that reversible methionine sulfoxidation plays in the assembly/disassembly of biomolecular condensates.

## 1. Introduction

Cells need to organize and coordinate their biochemical processes both in space and time to adapt to their changing environments. In this regard, compartmentalization has long been recognized as an important strategy of cellular regulation [1,2]. Even the simplest eukaryotic cell exhibits an intricate endomembrane system that defines compartments, also known as organelles, in which specific biological processes take place [3]. Using semipermeable membranes to divide the cellular space helps to separate chemically heterogeneous environments, providing optimal conditions for specific sets of reactions and avoiding potential interferences between biological processes that, in this way, can take place simultaneously. This is also true for organelles that are not part of the endomembrane system, such as mitochondria, plastids, and peroxisomes. Nevertheless, despite the advantages of a membranous system of compartmentalization, this regulatory principle is not a universal feature of life. Although some bacteria possess compartments bound by either a lipid bilayer (such as magnetosomes and thylakoids) or a lipid monolayer (lipid bodies) [4], the use of a proper endomembrane system to organize cellular functions is limited to eukaryotic cells.

**Figure 1:**
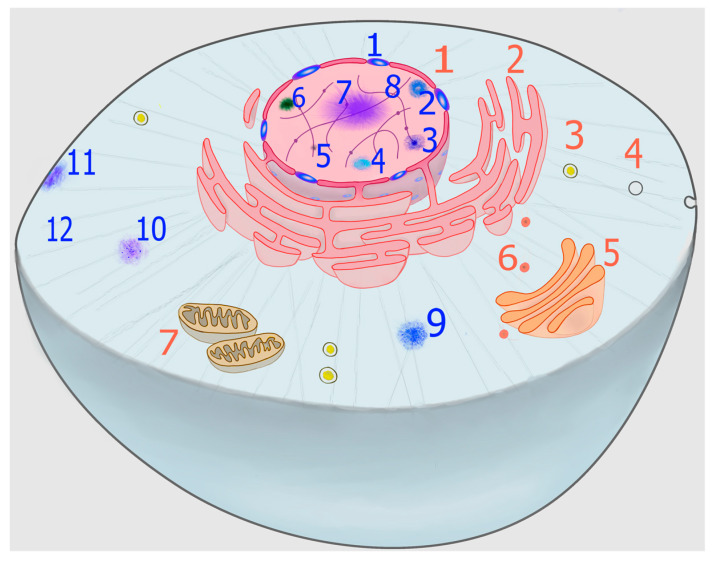
Cellular compartmentalization in eukaryotic cells. The main membranous organelles such as the nucleus (1), endoplasmic reticulum (2), lysosomes (3), endosomes (4), Golgi apparatus (5), vesicles (6), and mitochondria (7) are schematized using warm colors. On the other hand, nuclear MLOs such as nuclear pore (1), nuclear germs (2), PML bodies (3), paraspeckles (4), transcription puffs (5), Cajal bodies (6), nucleolus (7), heterochromatin (8), as well as extranuclear MLOs such as P bodies (9), SGs (10), membrane clusters (11), and cytoskeleton (12) are depicted in cold colors. The list of cellular compartments depicted here is not exhaustive. Some compartments occur only in specific cell types (i.e., synaptic densities and RNA transport granules in neurons; germ granules in germ cells; dicing bodies and photobodies in plant cells; carboxysomes in autotrophic bacteria, or pyrenoids in algae) and they are not shown in the figure.

In addition to membranous organelles, a new level of compartmentalization has gained conspicuous relevance in recent years, namely the formation of biomolecular condensates, which can be organized into specific but non-stoichiometric supramolecular structures that are generally referred to as membraneless organelles (MLOs) (Figure 1). Some of these structures, such as nucleoli, Cajal bodies, and nuclear speckles, were first described more than a century ago [5,6,7] and, although dynamic structures, they are stable enough as to allow their isolation by subcellular fractionation techniques [8]. Others, such as stress granules (SGs) and processing bodies (PBs), have been described more recently and are highly dynamic structures, rapidly condensing or dissolving in response to a wide range of stimuli. Therefore, they are ideally suited to be involved in rapid cellular adaptation to sudden stresses [9], as well as to metabolic changes associated with normal growth [10]. 

This battery of MLOs formed by biomolecular condensates is now understood to form via liquid-liquid phase separation (LLPS) of key protein and nucleic acid scaffolds, which recruit and retain, in a specific and regulated way, a wide and varied number of macromolecules (proteins and nucleic acids) that concentrate within the condensate and are referred to as resident clients [11]. Thus, LLPS endows the cell with an organizing principle [12], which allows compartmentalization on an optimal spatiotemporal scale for rapid adaptive responses to the cellular needs of the moment [9,13,14]. In contrast to classic membrane-based organelles, biomolecular condensates contribute to the compartmentalization of cellular processes in both eukaryotic and prokaryotic cells [4,15,16,17,18]. Although most research in LLPS has been focused on yeast and animal systems, in recent years there has been an increasing interest in the fields of plant science [19,20] and virology [21,22,23,24]. Plants are sessile organisms that are subjected to seasonal variance in environmental stressors, facing unique challenges during their life cycles. Therefore, in addition to MLOs that are shared between plants and other organisms, a number of biomolecular condensates that appear to be plant-specific have been described [19,20]. On the other hand, proteins of viral origin are also known to undergo LLPS. In a recent review about the relevance of LLPS in viral life cycle, Brocca and coworkers survey available data on LLPS of viral proteins, distinguishing LLPS related to viral replication and trafficking of viral components from virus-mediated LLPS processes interfering with host cell functions [23].

Since biomolecular condensates formed by demixing via LLPS seem to be universal, present in the three main domains of life, as well as in viruses, they likely represent the first compartmentalization strategy emergent in life history, driving the subsequent evolution of cellular complexity. In this regard, findings from research fueled by the recent interest in LLPS support the idea, put forward by Alexander Oparin more than nine decades ago, that life originated as coacervate drops of biomolecules accumulated in the primitive oceans [25]. 

Despite the stunning variety of MLOs described in the literature (Figure 1), it is important to identify unifying principles determining their formation, structure, and function. To this respect, the ability of the so-called scaffold macromolecules to form transient productive interactions (quinary structure) is of paramount importance [26]. This review aims to highlight some of these molecular underpinnings, with particular emphasis on the role that methionine residues from scaffold proteins can play in some cases.

## 2. Phase Transition: A Spontaneous Process Harnessed by Living Matter

Liquid-liquid phase transition is the process through which a solution demixes into two distinct solutions phases, a dense phase and a dilute phase that coexist stably with each other. The dense phase that is enriched in macromolecular solutes (proteins and/or nucleic acids) often resembles liquid droplets. This process is also often referred to as liquid demixing, coacervation or simply condensation (see [11] for a precise definition of the most used terminology in the LLPS field). Phase separation occurs when the macromolecule–macromolecule and solvent–solvent interactions are energetically favored over the macromolecule–solvent interactions (herein, the concept of solvent should be extended to include solutes other than the macromolecules). In this context, LLPS is determined by the balance between two opposite contributions to the free energy of the process: the loss of configurational entropy and the favorable enthalpic term that arises when intrachain interactions are exchanged for interchain interactions, which happens after the concentration of scaffold macromolecules has reached a threshold value [27,28]. On the other hand, the effect of macromolecules on the solvent properties and the resulting interfacial tensions are important factors that, to some extent, have been overlooked and can provide valuable insights into the LLPS behavior of biological systems [29,30].

Due to an avalanche of recent high-impact publications, a long renowned phenomenon known as macromolecular coacervation has moved into the focus of interest. Thus, LLPS, as a cellular mechanism to organize the cellular space and timing, has gained widespread attention across many fields in recent years. However, the observation that proteins tend to coacervate is not novel at all [25]. Since the seventies, it has been known that many enzymatic proteins can reversibly form high-order aggregates in response to changes in the concentration of their effectors [31,32]. Thus, citrate, an allosteric activator of acetyl-CoA carboxylase, induces the polymerization of an inactive protomeric form of the enzyme into an active filamentous form [32,33]. Similarly, mammalian glutaminase, a mainly mitochondrial enzyme [34,35] that can be targeted to the nucleus [36], is known to undergo high order oligomerization with concomitant changes in kinetic properties. Again, these changes are controlled by effectors (both activators and inhibitors) of this enzyme [31,37,38]. Although self-association does not necessarily imply a phase separation process, it has been demonstrated that oligomeric peptides undergo LLPS in vitro, with this process being stimulated by low temperatures, crowding agents, high protein concentrations, and pH close to the isoelectric point of the peptide [39]. In any event, the observations regarding acetyl-CoA carboxylase and glutaminase highlight two interesting facts: (i) proteins can readily alternate between different aggregation states and (ii) this dynamic alternation can be exploited to fulfil a regulatory role.

On the other hand, since the seventies we have known that glycolytic enzymes interchange between soluble and particulate states depending on different metabolic conditions [40,41,42,43], suggesting transient interactions that could result in an operative organization of glycolysis at the subcellular level [44]. Since then, the evidence that metabolic enzymes can coacervate has been increasing [45,46], leading to the concept of metabolon [47], defined as a transient structural–functional complex formed by sequential enzymes of a metabolic pathway held together by non-covalent interactions. Yet, the biological relevance of the hypothesized metabolon has remained controversial, mainly because of the lack of studies examining the dynamic distribution of these enzymes in vivo. However, very recently Colón-Ramos and coworkers, using a hybrid microfluidic-hydrogel device and *Caenorhabditis elegans* as model organism, have proven that phosphofructokinase, that is diffusely localized in the cytosol, can dynamically re-localize into biomolecular condensates in response to transient energy stress [48]. They further determined that these condensates formed in vivo exhibited liquid-like properties, including drop-like shapes due to surface tension, fluidity due to deformations, and fast internal rearrangements, overall suggesting LLPS as the mechanism underlying the formation of these structures, which were previously ruled out to be SGs by the authors of this report [48]. Of interest, a systematic study examining hundreds of yeast metabolic enzymes involved in intermediary metabolism identified the widespread reorganization of these proteins into reversible assemblies upon nutrient starvation [49], suggesting that dynamic compartmentalization into condensates via LLPS could represent an extended regulatory strategy.

The experience of crystallographers, who often observe the phase separation of proteins as a side product of the crystallization process [50,51], also lends support to the view that many, if not all, proteins have an intrinsic ability to undergo phase separation that is manifested in conducive conditions [52]. The ability to be engaged in LLPS may be a universal property of proteins under specific conditions, but for many proteins, such conditions may never be encountered in a cell. That is to say, LLPS may be accessible to a subset of proteins under the conditions that exist within living cells [53]. Thus, identifying the common features of proteins that experience LLPS in a biologically meaningful manner is of paramount importance. In recent years, impressive progress has been made towards understanding the molecular signatures present in molecules that can phase separate under physiological conditions. A brief discussion of this progress is presented next. 

## 3. Molecular Determinants and Modulators of LLPS

We have defined phase separation as the process through which a solution transitions from a homogenous state to a demixed two-phase system with lower Gibbs free energy. Characterization of these systems via the empirical construction of phase diagrams, is an approach that has allowed to obtain important insights regarding the molecular determinants that promote the phase separation of scaffold proteins, as well as the conditions (pH, temperature, ionic strength, or macromolecular concentration) that are compatible with LLPS. 

### 3.1. Concentration of Macromolecules

**Figure 2:**
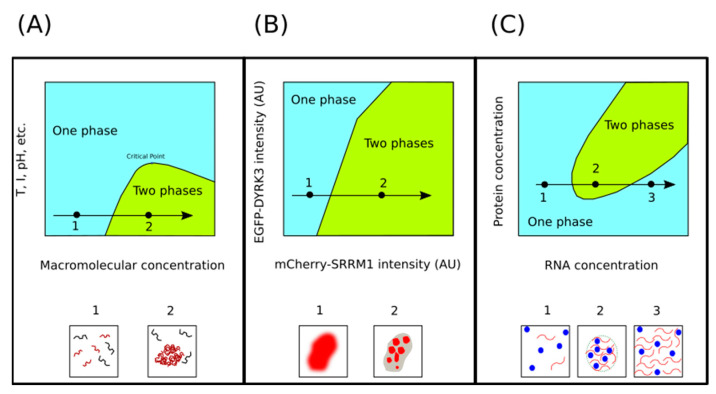
Schematic phase diagrams; (**A**) The black curve separating the plane into two regions (one-phase in cyan and two-phase in green) informs about the conditions (macromolecular concentration; temperature, T; ionic strength, I; pH; etc.) at which the two regimes coexist in equilibrium. At a given value of the environmental variable, an increase in the concentration (black arrow) of the scaffold macromolecule leads from the one-phase to the two-phase state (squares 1 and 2, respectively, where the scaffold macromolecules have been represented in red). (**B**) Schematic representation of the results reported in [55]. In a population of cells expressing the fluorescent tagged proteins EGFP-DYRK3 (a kinase with dissolvase activity) and mCherry-SSRM1 (a nuclear substrate of the DYRK3 kinase), nuclear intensities of both signals can be recorded and plotted in a Cartesian plane using arbitrary units (AU). The black line splitting the plane in two regions is given by the kinase/substrate ratio above which the nuclear condensates containing SSRM1 melt. Thus, at a fixed value of kinase concentration, moving from lower to higher concentrations of the kinase substrate (black arrow) leads to the assembly of condensates (square 1 and 2, respectively). (**C**) Phase diagram of RNA-protein mixtures. The black arrow represents, at a given protein concentration, the direction in increasing RNA concentrations. An initial increase in RNA concentration drives phase transition from one phase (square 1) at low RNA levels to two phases (square 2) at intermediate levels of RNA. Further increasing the RNA concentration drives the second phase transition, now from two phases (square 2) to one phase (square 3) after dissolution of the condensates. This process, known as RNA-mediated reentrant phase transition, is thought to be driven by electrostatic forces.

Phase transitions are extremely sensitive to macromolecular (protein and nucleic acids) concentration. Within a range of temperature, pH, and salt concentration values, increasing macromolecular concentration leads to demixing (Figure 2). The basis for this observation is that, at high macromolecular concentration, the decreasing free energy due to intermolecular interactions overcompensates for the decreasing configurational entropy. However, this stabilizing contribution is insufficient to compensate for the decreasing configurational entropy unless the macromolecular concentration reaches a threshold value [28]. Furthermore, in some circumstances, it is also important that the scaffold components do not exceed a second, and higher, threshold concentration value [54]. In any case, we are interested in understanding how cells can exploit this concentration-sensitivity to regulate LLPS in a biologically meaningful manner. To illustrate this point, we shall present two examples from very different contexts.

Upon entering mitosis, many MLOs melt and then later, during telophase or after the completion of mitosis, reappear again [56,57]. Experiments carried out using human cells suggest that the timing of these LLPS processes is regulated by the dual specificity tyrosine-phosphorylation-regulated kinase 3 (DYRK3), a protein kinase that acts as a dissolvase of MLOs during the G2 to M transition, after the nuclear envelope breakdown [55]. If upon entry into mitosis MLOs disassembly occurs via DYRK3, one could naively hypothesize that its dissolvase activity should suddenly increase at the beginning of mitosis and then reduce at the end of mitosis. However, Rai and collaborators did not observe such a sudden increase in DYRK3 levels as cell entered mitosis [55]. Thus, to explain how DYRK3 drives the disassembly of MLOs during mitosis, these authors noted that upon breakdown of the nuclear envelope, the concentration of DYRK3 relative to its substrates suddenly increase. That is possible because key substrates of DYRK3 are exclusively located in either the nucleus (serine/arginine repetitive matrix protein 1, SRRM1) or the cytoplasm (pericentriolar material 1 protein, PCM1), while DYRK3 itself is found in both compartments. Thus, when the cell enters mitosis and the nuclear barrier is removed, the concentrations of the DYRK3 substrates, but not that of the kinase, are diluted. In line with this explanation, these authors observed a sharp phase boundary in their phase diagrams, at a specific DYRK3-to-SRRM1 ratio, below which SRRM1 would be condensed and above which it would be dissolved (Figure 2B).

The other example, illustrating the effect of macromolecular concentrations on condensate formation, comes from the transcriptional regulation field. Thus, in a recent paper, Henninger and coworkers describe a non-equilibrium feedback control mechanism for the regulation of transcription. According to the proposed model, at a low concentration of nascent RNAs, electrostatic interactions promote the formation of transcriptional condensates, whereas the burst of RNAs produced during elongations trigger the condensate dissolution (Figure 2C), providing a feedback mechanism [58]. This phenomenon where RNA at low concentration drives the formation of droplets, while high RNA concentrations lead to the dissolution of these droplets, is referred to as reentrant phase transition (RPT) [54]. An electrostatic mechanism seems to be behind RPT. Indeed, many RNA-binding proteins that undergo LLPS present intrinsically disordered regions (IDRs) enriched in basic (arginine and lysine) residues [59]. Therefore, when these proteins are in contact with low RNA concentrations, short-range electrostatic attractions lead to charge neutrality, favoring the formation of biomolecular condensates. However, when RNA is found at high concentrations, long-range Coulombic repulsion derived from a charge inversion will drive the dissolution of these condensates [60,61].

### 3.2. Multivalency

LLPS of scaffold proteins and the subsequent partitioning of clients to form MLOs rests on the establishment of a network of intermolecular interactions. Therefore, scaffold proteins must be equipped with a domain architecture that facilitates such interactions. Two archetypes of protein architectures optimal to promote interaction networks have been distinguished, both having multivalency in common. That is, the protein presents multiple motifs able to interact with their partners [53].

The first archetype consists of multiple structured domains, such as SH2 and SH3, that interact with short linear motifs in their partner proteins, such as phosphotyrosine or proline-rich motifs, respectively. The role that these folded domains can play in LLPS has been smartly documented by Li and coworkers, who generated two classes of engineered proteins, one composed of repeats of a single SH3 domain (SH3_m_, were m = 1–5) and the other composed of repeats of proline-rich motifs (PRM_n_, where *n* = 1–5) [62]. Using different combinations of these engineered proteins, they observed LLPS, whose phase boundary was strongly dependent on the valency of the mixed proteins. That is, the concentrations needed for phase transition were directly related to the valency of the interacting species (SH3_m_-PRM_n_). Of interest, this behavior observed in vitro was mirrored in living cells. Thus, HeLa cells co-expressing mCherry-SH3_5_ and eGFP-PRM_5_ formed cytoplasmic foci containing both fluorophores. However, these condensates were not observed in cells expressing either protein alone or in cells co-expressing fusion proteins with lower valences [62].

The second archetype is characterized by the presence of RNA recognition motifs (RRMs) as well as intrinsically disordered regions (IDRs) of low complexity (LC) [63]. These domains show biased amino acid compositions. The amino acid composition of these domains and their disposition in the modular architecture of the scaffold proteins determine the networks of weak but cooperative interactions that can be formed, which in turn govern the LLPS process [64]. Of note, the sort of interactions critical for LLPS are of the same nature as the interactions that drive protein folding and stabilize the tertiary structure of proteins [65,66]. Thus, LC domains rich in aromatic residues (tyrosine and phenylalanine) are favored to form π−π stacking interactions [64,67]. Although π−π interactions are commonly associated with aromatic rings, π orbitals of bonded sp^2^-hybridized atoms, as those found in peptide backbone amide groups, may also be involved in stabilizing interactions. In this regard, residues such as glycine, serine, threonine, and proline (often found to be overrepresented in LC regions) have small side chains. This reduced size implies relatively exposed backbone peptide bonds enabling planar π interactions that can contribute to phase separation [68].

On the other hand, RRMs are enriched in basic amino acids that facilitate the binding of negatively charged RNA molecules, but these basic residues (mainly arginine [69]) can also interact with aromatic amino acids to form cation-π interactions, which have been shown to be critical in modulating FUS (fused in sarcoma) phase separation [64,70]. In addition to mediate protein-nucleic acid binding, charge-charge interactions are also relevant in protein-protein interactions, playing a prominent role in the phase separation of certain scaffold proteins. For instance, DEAD-box helicase 4 (Ddx4), a protein essential for the assembly and maintenance of the related nuage in mammals, contains alternating clusters of net negative and net positive charge. To address the importance of this charge distribution in phase separation, Nott et al. constructed a Ddx4 mutant, with the same overall net charge but in which the clusters were scrambled. The mutated proteins were unable to support LLPS [71]. 

Hydrogen bonds also can be important in driving phase separation. All amino acids may participate in hydrogen bonding, either as hydrogen bond donors or acceptors. However, the most prone are polar ones. Indeed, NMR and simulation techniques have shown that glutamine residues abundant in the LC region of FUS are important determinants of phase separation, being hydrogen bonding highly prevalent [72].

In general, what interactions contribute proportionally more to LLPS, and how these interactions may be tuned by environmental conditions, will depend on the specific amino acid composition of a given scaffold protein. In other words, different proteins may resort to different combinations of interactions, thus enabling a selective regulation. To illustrate this point, later, we will focus our attention on a group of scaffold proteins whose LC regions contain an unusually high frequency of methionine [14,73,74] Methionine is an amino acid known to participate in a number of non-covalent bonds, some of them highly specific for this amino acid [75,76], that overall contribute to protein stability. Therefore, methionine-dependent interactions may be key elements in the LLPS process of this group of proteins. Nevertheless, since the role of methionine in LLPS is a subject of particular interest in the current review, we will address its discussion in more detail in a subsequent section.

### 3.3. Intrinsic Disorder

In the preceding section we have introduced the concept of intrinsically disordered regions in the context of multivalency. Now, in the current section we would like to emphasize the importance of intrinsic disorder in phase separation. To this end, we will briefly summarize the role that IDRs play not only as drivers of the LLPS process, but also as key regulators of the composition, stability, and functionality of the diverse MLOs that can be formed within cells.

IDRs constitute a significant fraction of any known proteome, with the percentage of residues from the whole proteome involved in disorder ranging from 12% to 36% [77]. What characterizes a protein, or a region of a protein, as intrinsically disordered is that it fails to form a unique, predominantly stable 3D-structure, yet IDRs fulfil many biologically important functions [78]. Their inability to adopt a fixed structure is encoded by their amino acid sequences. Although IDRs can appear in different flavors, depending on the amino acid composition bias exhibited [79], in general they are deficient in canonical hydrophobic residues, which are core residues for folded domains, but are enriched in structure-breaking amino acids that make IDRs flexible enough to dynamically adopt different conformations that populate a flat energy landscape. As we will argue later on, this high conformational heterogeneity is critical to the role these proteins play in MLO biology.

An overwhelming number of different MLOs have been described in the literature, and the list keeps growing as new cellular biomolecular condensates continue to be discovered on a regular basis [77]. These MLOs come in varied sizes and shapes, they have different composition, and they show specific biophysical and biochemical properties, likely reflecting the requirements of the different cellular processes in which they participate, as well as the specific regulatory needs of these processes. However, a common denominator is that almost invariantly all these MLOs contain proteins with IDRs [77]. This observation raises a question: What are the features of IDRs that make them so relevant in the biology of MLOs?

An obvious explanation, that we have already discussed in the previous section, is that IDRs contribute to the multivalency phenomenon via their tailored LC regions. Furthermore, the conformational flexibility exhibited by IDRs allows the interacting residues (stickers) to be spatially disposed to yield productive bindings while avoiding unspecific contacts, which is particularly important in the context of an overcrowded milieu as is the case of biomolecular condensates [77]. Another important feature of IDRs is that they usually cluster amino acids susceptible to post-translational modifications (PTMs). Indeed, the lack of a compact 3D-structure facilitates the accessibility of the target sites to various PTMs. As will be discussed in a subsequent section, PTM is relevant to the regulation of LLPS.

A further interesting consideration, regarding the suitability of IDRs as MLOs constituents, is their contribution to the stability and resilience of the supramolecular structures of which they are part. Despite the lack of membranes and the fact that their constituents are freely exchanged with the environment, MLOs are stable entities. As paradoxical as it may sound, Uversky postulates that the lack of structure in IDRs is what determines the stability and resilience of cellular condensates [80]. This author argues that “there is a remarkable difference in the resilience of rigid complexes formed by rigid blocks and stabilized via the specific and high affinity block–block interactions and fluid complexes made of flexible constituents”. In other words, while structured supramolecular complexes are much less resistant to the loss of any of their components, biomolecular condensates can either gain or lose IDRs components without drastic changes in the overall stability of the condensate.

### 3.4. Transient Secondary Structural Elements

Apart from amino acid side chain interactions, LLPS can be influenced by secondary structural elements [81,82]. Within the LC region of scaffold proteins, stretches of amino acids can assemble into intermolecular cross-β sheets resembling those typically found in amyloid fibrils [83]. However, structural studies of fibrils formed by short segments of the LC regions from proteins that undergo LLPS, have uncovered important differences with classic amyloid fibrils. Thus, while amyloid fibrils tend to have cross-β sheets with interdigitated amino acids that form steric zippers, those fibrils formed by short segments of the LC region of proteins, being phase separated, have kinked cross-β sheets, and are referred to as low-complexity aromatic rich kinked segments (LARKS) [84]. Unlike steric zippers of amyloid fibrils, the kinked sheets are thermodynamically less stable, providing a reversible force for the assembly/disassembly of biomolecular condensates [85].

## 4. Posttranslational Modifications Others than Methionine Sulfoxidation

Life uses a common set of 20 coded amino acids to build proteins. The physicochemical diversity covered by this set of proteinogenic amino acids can be extended further through PTMs of the side chain of most of these amino acids. More than 400 different PTMs have been described [86]. Often, PTM involves the addition of chemical functional groups to the side chain of target residues. These modifications range from the addition of a single atom (e.g., sulfoxidation of methionine) to the addition of a whole polypeptide chain (e.g., ubiquitination of lysine). In other cases, PTMs involve the modification of the chemical properties of the target amino acid by replacing one chemical functional group with another. Thus, the deamidation of glutamine and asparagine or the deimination of arginine (also called citrullination) are examples belonging to this last category.

Each of these PTMs has, independently, the potential to regulate the activity of the modified protein, its subcellular location, its stability (folding), its affinity for other cellular components, etc. In addition, in recent years, the possibility of interrelationships between different PTMs (cross-talk) has begun to generate great interest [87]. These interrelations allow an enormous expansion of the degree of regulation and the number of functional states that a single protein can reach. Thus, combining multiple PTMs, a single protein can yield a large number of proteoforms [88], which has led to the concept of PTM code [89]. In summary, because of the potential to elicit an additive, cooperative, or competitive integrated response [90], the PTM code can provide many layers of complex biological regulations that are not yet well understood.

Because of their accessibility to modifying enzymes, IDRs found in scaffold proteins are the predominant sites of PTMs [91]. Since LLPS is driven by a network of residue-to-residue interactions, modifications of these residues from IDR can alter, either increasing or weakening, the network of interactions. As we have seen above, arginine is a key amino acid involved in LLPS-driving interactions. Indeed, this basic residue can take part in (i) cation-π bonds, (ii) salt bridges, (iii) or participate in the binding of nucleic acids. In addition, arginine specifically binds ATP, which biphasically modulates LLPS of the scaffold protein TDP-43 [92]. Not surprisingly, arginine modification by members of the protein arginine methyltransferase (PRMT) family plays a suppressive role in phase separation [70,71,93,94]. PRMTs catalyze the transfer of one or two methyl groups from S-adenosylmethionine to the nitrogen atoms of the guanidinium group of arginine [95]. Interestingly, the cellular concentration of S-adenosylmethionine has been proposed as a regulator of SG composition and assembly [96]. Although arginine methylation is thought to reduce LLPS, this modification may fulfil more complex regulatory roles, affecting the dynamics and functional properties of biomolecular condensates in numerous and different ways [97].

Beside arginine methylation, citrullination, the conversion of arginine into citrulline, is another PTM that targets arginine residues and can impact the LLPS process [98]. This conversion is catalyzed by the peptidyl arginine deiminase (PAD) enzyme family, which replaces the imine group of arginine by a carbonyl group [99]. In this way, the positively charge guanidinium group, -NH-C(NH_2_)_2_^+^, is transformed into a neutral urea group (-NH-CO-NH_2_). It seems that arginine citrullination reduces the multivalent interactions between tyrosine and arginine residues and hence has a inhibitory effect on LLPS [97].

Unlike arginine methylation, considered a rather stable modification [95,100], phosphorylation of serine, threonine and tyrosine residues is a highly dynamic PTM playing a relevant role in the control of biomolecular condensates (reviewed in [90,97]). Replacing the neutral hydroxyl group (-OH) of the phosphoacceptor residue with a bulky phosphoryl group (-PO_4_^2^) entails a drastic change of the steric, chemical, and electrostatic properties of the affected side chain, introducing new interaction capabilities. Thus, the introduced negative charges can form strong salt bridges, mainly with arginine residues due to the rigid planar structure of the guanidinium group, and its ability to form multiple hydrogen bonds [101]. Phosphorylation can also affect the stability of secondary structure elements, either increasing or decreasing them. Thus, phosphoserine located at the N-terminal positions of the helix has a stabilizing effect, while it has a destabilizing repercussion in the helix interior [102]. Phosphorylation may also interfere with the hydrogen bonding affecting the formation of cross-β structures. That has been suggested to be the cause of the reduced phase separation of the scaffold protein FUS after phosphorylation by DNA-PK of serine and threonine residues from its LC region [97].

In contrast to FUS, where phosphorylation has a suppressive effect on phase separation, a high degree of tyrosine phosphorylation on the IDR cytoplasmic tail of nephrin promotes the phase transition in the assembly of multivalent signaling aggregates [62]. Nephrin has six potential phosphotyrosine sites, each of which can interact with SH2 domains from NCK, an adaptor protein. In addition to SH2 domains, NCK also has SH3 domains able to interact with six proline-rich motifs of N-WASP, a protein that regulates actin polymerization by stimulating the actin-nucleating activity of the Arp2/3 complex [103]. Tau, a neuron-specific protein, also illustrates how phosphorylation can favor phase separation. Thus, serine phosphorylation in the microtubule binding domain of Tau, which is lysine-rich, drives electrostatic coacervation promoting LLPS [104].

Other PTM that appears to be involved in the regulation of LLPS is acetylation of lysine residues. Thus, this modification has been shown to decrease phase separation of the proteins Tau [105,106] and RNA helicase DDX3X [107], a component of SGs. Furthermore, the deacetylase enzyme HDAC6 seems to be required for SG formation [108]. Likewise, O-GlcNAcylation of serine and threonine residues has been described to cross-talk with phosphorylation in Tau [109] and hnRNPA1 [110]. In summary, although the role of cross-talk between different PTMs in the control of LLPS is currently ill defined, it can be anticipated that future work to disentangle the PTM code will provide interesting insights regarding the dynamics and regulation of MLOs formation.

## 5. Role of Methionine Residues in Modulating LLPS

The oxidation of protein-bound methionine to form methionine sulfoxide (MetO) has traditionally been regarded as an undesirable and harmful consequence of oxidative stress, linked to age-related malfunctions [111,112,113,114]. More recent works have allowed to broaden and enrich this view. Thus, investigations from a number of laboratories support the notion that reversible protein methionine sulfoxidation can act as an antioxidant buffer system [115,116,117,118]. In addition, methionine oxidation activates transcription factors in response to stimuli [119,120], regulates the cytoskeleton dynamics [121,122,123], and the activity of key signaling protein kinases [124,125], just to mention a few examples. The interconversion of Met and MetO also influences protein stability and protein-protein interactions (PPI) [76,126]. A more comprehensive information regarding the impact on biology of methionine oxidation can be consulted in the database MetOSite, a resource devoted to this end [127]. Although the oxidation of methionine residues has been reported to have multiple and varied implications for protein function, in the remaining sections of this article we will focus on very recent findings that identify reversible methionine oxidation as a redox sensor involved in the dynamic assembly/disassembly of biomolecular condensates. These studies represent a breakthrough, as they provide insight into how MLOs can act as coupling agents that sense the metabolic state of the cell and develop appropriate adaptive responses [14,73,74,128]. 

### 5.1. Short Overview on Methionine Properties Relevant for LLPS

Methionine, an aliphatic sulfur-containing molecule, is endowed with physicochemical properties that make it a unique proteinogenic amino acid. Next, we point out some of these properties that could be relevant to understand the role of methionine residues in LLPS and its regulation. A more detailed description of these properties and the discussion of their relevance in a wider biological context can be found at [129].

Although methionine is considered a hydrophobic amino acid, unlike other hydrophobic residues such as leucine, isoleucine or valine, methionine has an unbranched side chain, which provides ample flexibility. In this way, when patches of methionine residues are arranged in the primary structure of a protein, the extra-flexibility of methionine provides a malleable nonpolar surface that can adapt itself to peptide binding partners, favoring the proximity between atoms from different polypeptide chains and the subsequent van der Waal’s interactions [130]. Furthermore, the larger polarizability of sulfur with respect to carbon also leads to greater London dispersion forces.

A third property that also contributes to the plasticity of methionine residues during PPIs is related to the $X_{3}$ torsion angle, which controls the position of the ε-methyl group. A statistical survey of a wide sample of proteins revealed that $X_{3}$ remained virtually flat over the entire range of possible values [131]. This observation implies that the side chain of methionine shows very little energetic preference between different conformations, which in turn confers to methionyl residues a wide freedom to mold themselves to accommodate binding partners. Overall, these properties make methionine stand out from the rest of the hydrophobic amino acids. Next, we present an intermolecular interaction of a very different nature to the weak van der Waals forces described above. This interaction, which is referred to as an S-aromatic bond, places the methionine sulfur atom at the center of the picture [132,133,134].

Indeed, a remarkable property derived from the presence of a sulfur atom in the side chain of methionine is the potential to interact with nearby aromatic residues, such as tyrosine, phenylalanine, and tryptophan, to form the so-called S-aromatic motif [75]. These motifs can computationally be identified on the basis of geometric considerations, including the interatomic distance between the sulfur atom of methionine and the centroid of the aromatic ring [135]. Thus, when this distance is below the threshold of 7 Å, the interaction can have an associated energy comparable to that of a salt bridge. However, the strength of these non-covalent bonds can vary dramatically depending on the environment around the sulfur [126,136,137], which may be exploited from a regulatory point of view, as we will discuss later.

Finally, the sulfur atom of methionine can be readily oxidized to form methionine sulfoxide (MetO). This oxidation reaction can be catalyzed by enzymes [122,123,125,138]. However, the number of proteins proved to be oxidized in an enzyme-catalyzed reaction is very small. Although we can anticipate that this list will be extended in the future, only three proteins are included in it, namely calmodulin [138,139], F-actin [122,123], and Ca^2+^/calmodulin-dependent protein kinase II (CaMKII) [125]. Nevertheless, hundreds of proteins are known to be sulfoxidized within living cells in response to oxidative stimuli [140]. Furthermore, many of these oxidation sites seem to be selectively targeted rather than randomly modified [141]. In any event, MetO can be reduced back to methionine in a reaction catalyzed by methionine sulfoxide reductases (Msrs), enzymes present in all eukaryotic cells and in most prokaryotic cells [142]. Therefore, the oxidation/reduction of methionyl residues represents a reversible post-translational covalent modification, particularly suitable for sensing the cellular redox state and mediating redox signaling [141,143].

### 5.2. Effects of Methionine Oxidation on Transient Protein-Protein Interactions

The simple covalent addition of an oxygen atom to the sulfur atom of methionine residue can cause drastic changes in the properties we have outlined above. These changes, in turn, can impact the physicochemical properties of the whole protein. Since PPIs are a relevant aspect of LLPS, we next focus on the influence of methionine sulfoxidation on these interactions.

Perhaps the most obvious change caused by the oxidation of methionine is the conversion of an apolar side chain into a highly polar one. Thus, the side chain hydrophobicity index decreases from 0.738 (Met) to 0.238 (MetO) after the incorporation of the oxygen atom [144]. This decrease in hydrophobicity is behind the rationale of using glutamine (hydrophobicity index of 0.251) in mutagenesis experiments to mimic the sulfoxidized state of a protein [119,139]. Here, a caveat is in order. Although glutamine and MetO exhibit a remarkable structural similarity and almost identical polarity, the amido group-containing glutamine still packs well into α-helices, in contrast to MetO [145]. In any case, whether it is due to a simultaneous increase in polarity and bulkiness while losing flexibility or due to a loss of secondary structure, methionine sulfoxidation seems to impair protein–protein interactions [73,74] more often than favoring them [126,146]. Thus, although the oxidation of methionine residues has been reported to favor the formation of cross-β amyloid structures, this behaviour is most likely a colateral effect subsequent to a partial unfolding of the protein [146].

We have previously underlined the importance of a flexible side chain for promoting close contact between two hydrophobic surfaces, each one belonging to one of the interacting proteins. However, if we aim to understand the role of methionine sulfoxidation in the control of PPI, we must first address two issues. On one hand, since the reactivity of protein-bound methionine against oxidants is strongly influenced by the accessibility of the sulfur atom [147], how is it possible to oxidize methionine residues that are forming part of these contact surfaces that are excluded from the medium? On the other hand, since we are considering transient PPIs, does it mean that the protein, in the absence of its partner, exposes large hydrophobic patches to the surrounding medium? Although, on the basis of theoretical considerations, different scenarios could be proposed to reconcile these problematic issues with a role for methionine modification in the regulation of PPI, we would rather limit ourselves to those explanations that seem to have an experimental support [74]. To this end, we next review in detail the work carried out with a few proteins that we could take as paradigms in the redox control of LLPS.

### 5.3. TDP-43

**Figure 3:**
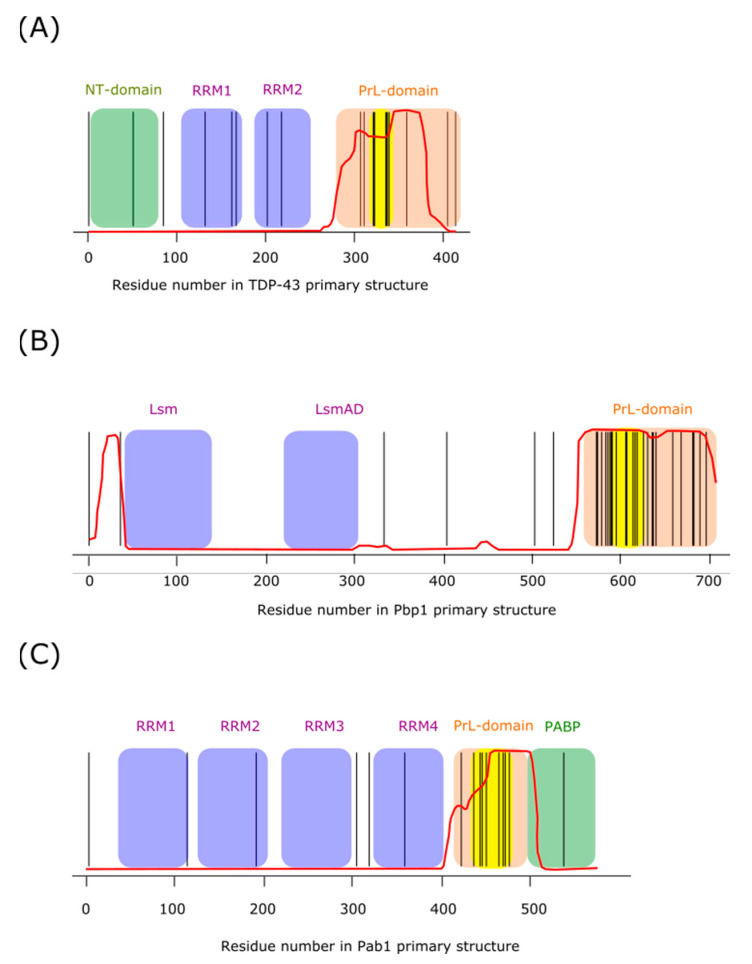
Modular architecture of three scaffold proteins containing methionine-rich LC domains. Methionine positions in the primary structure are indicated by vertical black lines. (**A**) TDP-43 presents a folded N-terminal domain (green box) with six β-strands [151], which has been shown to play an important role in the aggregation of TDP-43 monomer. This protein also has two RRMs (blue boxes) and a PrL domain (salmon box) that host a redox sensor formed by methionine residues (yellow box). (**B**) Pbp1, the yeast ataxin-2 orthologous, exhibits two RNA binding domain, Lsm and LsmAD, (blue boxes) and one methionine-rich LC regions (salmon box) containing a redox sensor (yellow box) that controls the aggregation ability of the protein. (**C**) Pab1 has 4 RRMs (blue boxes) one PrL domain (salmon box) enriched in methionine residues (yellow box) and a poly(A)-binding protein (PABP) domain towards the C-terminal of the polypeptide chain (green box). The red curves represent the score provided by the software PLAAC for each protein sequence. PLAAC uses Hidden Markov Models to compute the probability of a region from the analyzed protein being an LC region belonging to the PrLD category [156].

TAR DNA-binding protein 43 (TDP-43) is a protein involved in RNA biogenesis and processing [148]. It has been implicated in the formation of neural granules, and contributes to localize dendritic translation at synapses [149]. The domain architecture of this protein (Figure 3A) contains a structured N-terminal domain that facilitates homotypic oligomerization [150,151], two RNA recognition motifs (RRM), and a low complexity (LC) region that is identified as a prion-like domain (PrLD). Both elements, RRM and PrLD, are features present in many proteins able to undergo LLPS, which are prone to misfolding and aggregation, processes linked to neurodegenerative diseases [63]. A number of mutations in the gene encoding TDP-43 have been related to neurodegenerative diseases [63,152]. Not surprisingly, in recent years, TDP-43 has emerged as one of the most intensively studied proteins in the field of neurodegenerative diseases. Despite all this research effort, we still have only an incomplete and fragmented view of the biochemistry of this protein. Nevertheless, we can point some interesting facts: (i) the vast majority of the disease-favoring mutations are found in the C-terminal PrLD of the protein [63,153], (ii) the PrLD is essential for recruitment of TDP-43 into SGs [154], (iii) studies done on recombinant purified TDP-43 suggest that a region within the PrLD might form the core of TDP-43 aggregation [155].

These observations have led to the view that PrLDs tend to behave as autonomous aggregation modules that, eventually, may adopt amyloid-like pathological conformations. However, based on recent findings, Franzmann and Alberti have proposed a rather opposite perspective. According to these authors, PrLDs may have evolved to regulate LLPS and to protect cells against proteotoxic damage [157]. The arguments given to support this picture are as follows. The folded domains of RNA-binding proteins, in order to carry out its functions must interact with its natural ligands, such as RNA, nucleotides, and other binding partners. These functional requirements may have imposed evolutionary constraints that make these domains highly prone to aggregation. In this scenario, PrLDs may act as regulatory elements that avoid aberrant behaviors of the whole protein by acting as modifiers of protein phase transitions that adjust the solubility of proteins and protect them from misfolding [157]. Hitherto, the description we have presented of the LC region of TDP-43 could easily be generalized to that of many other RNA-binding proteins containing PrLDs. Now, we will focus on the peculiarities of the TDP-43 PrLD.

The PrLDs of many RNA-binding proteins are characterized by a high abundancy of tyrosine and/or phenylalanine. The cation–π interaction established between these aromatic residues from the PrLD and arginine residues from the RRMs, has been proved to be determinant in setting the protein saturation concentration during LLPS [64]. In contrast, the LC domain of human TDP-43 only contains one tyrosine and five phenylalanine residues. However, in addition, it departs from prototypic PrLDs in the presence of 10 evolutionary conserved methionine residues expanding from residue 307 to 414 from the human sequence [74]. Although there exists certain controversy with regard to whether this protein region could be involved or not in the formation of cross-β assemblies during LLPS in vivo [85,158], McKnight and coworkers have recently reported convincing arguments in favor of the view that labile cross-β structures may be responsible for the formation of droplets in vitro, as well as condensates within living cells. Furthermore, these authors also delineate the key role played by methionine residues both in the formation of the aggregates and in sensing the conditions leading to their disassembly [74].

A first experimental indication that methionine residues from the LC region of TDP-43 may play a key role during condensation came from in vitro experiment with purified protein. Thus, the authors expressed recombinant proteins containing the terminal 152 residues of TDP-43 fused to different tags. When these proteins were incubated under physiological conditions (regarding monovalent salt concentrations and pH), the solutions were able to transit into a gel-like state that, when evaluated by X-ray diffraction, yielded prominent cross-β diffraction rings at 4.7 and 10 $\dot{A}$. Prior to gelation, light microscopic examination of the solutions revealed droplets with diameters ranging from 2 to 10 μm. Interestingly, when the observed droplets were exposed to varying concentrations of hydrogen peroxide (H_2_O_2_), a progressive melting of the droplets was observed until droplets fully disappeared at 0.3% H_2_O_2_. By contrast, when the LC region of TDP-43 was substituted by that of FUS, another RNA-binding protein that undergoes LLPS but that only contains a single methionine residue in its LC domain, no evidence of melting could be obtained even upon exposure to 1% H_2_O_2_. In line with the hypothesis that droplet melting was caused by the oxidation of methionine residues, droplet reformation was observed upon the addition of a mixture of MsrA, MsrB, thioredoxin, thioredoxin reductase, and NADPH. Msrs enzymes are known to reduce MetO back to methionine in the presence of thioredoxin, thioredoxin reductase and NADPH [159]. Furthermore, direct evidence of the H_2_O_2_-driven methionine oxidation of the TDP-43 PrLD was provided by mass spectrometry [74].

**Figure 4:**
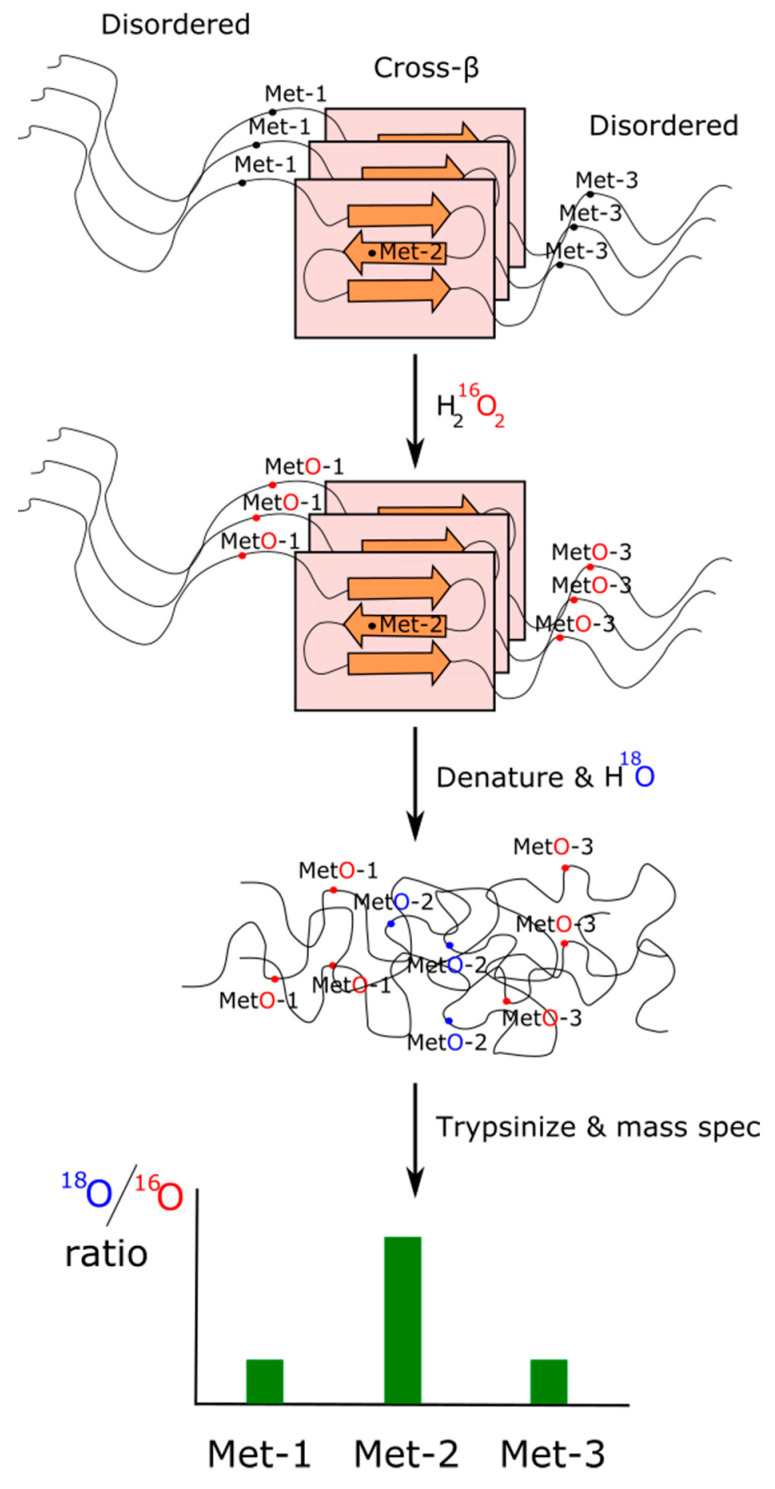
Schematic representation of H_2_O_2_-based footprinting of proteins. Three identical polypeptide chains, interacting by mean of a cross-β structure, are represented. The N- and C-terminal ends of each chain are supposed to be disordered and contain accessible methionines (Met-1 and Met-3). The central β-sheets also contain methionine (Met-2) but this residue is not accessible as it is protected by the laminated β-sheets that form the cross-β structure. The protein is initially exposed to limiting amounts of ^16^O-labeled H_2_O_2_, which will oxidize those methionines being accessible (MetO-1 and MetO-3). Afterwards, the protein is denatured and oxidized to completion with ^18^O-labeled H_2_O_2_, allowing the oxidation of Met-2 to MetO-2. Finally, samples are analyzed by mass spectrometry to determine the ^18^O/^16^O ratio of each methionine residue. This figure is a modification of the scheme found in the supplementary materials accompanying the paper [74].

As we have pointed out in the previous subsection, those methionines forming part of a structured contact surface are expected to exhibit a lower reactivity against oxidants than those that are more accessible to the solvent [147]. Based on this rationale and using an isotopic labeling strategy described elsewhere [160], Lin and coworkers designed a method of H_2_O_2_-mediated footprinting that they applied to characterize cross-β polymers formed from the TDP-43 PrLD. Briefly, polymeric forms of the TDP-43 PrLD were exposed to limiting concentrations of ^16^O-labeled H_2_O_2_ to allow the oxidation of only those methionine residues being more exposed/reactive. Afterward, the excess of oxidant was quenched, and the desalted protein was denatured by treatment with 6 M guanidine HCl. Completely unstructured protein was then exposed to ^18^O-labeled H_2_O_2_. In this way, those methionine residues that remained unoxidized because they were protected while forming part of a cross-β structure should now be exposed and oxidized. Therefore, methionines involved into the formation of cross-β polymers should show a high ^18^O/^16^O ratio when analyzed by mass spectrometry (Figure 4). Using this elegant experimental design, the footprint of TDP-43 in liquid-like droplets showed Met322 and Met323 to be substantially protected from the oxidant, which points to these residues as integrant of the cross-β core. A parallel result was obtained when the oxidation with ^16^O-labeled H_2_O_2_ took place within living cells and then solubilized and immunoprecipitated TDP-43 was treated with ^18^O-labeled H_2_O_2_. Again, Met322 and Met323 showed a much higher ^18^O/^16^O ratio, suggesting that they form part of a structured segment of the protein, most likely a cross-β structure [161]. As a note of interest, multiple sequence alignment of 11 vertebrate species ranging from fish to human reveals that the 23 residue long segment ^319^NPAMMAAAQAALQSSWGMMGMLA^341^ (numeration corresponding to the human sequence) remained conserved after the last common ancestor split some 500 million years ago [74]. Interestingly, this segment also coincides with a cross-β structure formed by segments of the TDP-43 protein characterized by cryo-EM [161]. Furthermore, mutations of methionine from this segment to either valine or isoleucine have been related to neurodegenerative diseases [63].

If we accept that oxidants arising inside cells, as consequence of their activities, control the assembly/disassembly of TDP-43 condensates, we must still answer the question we posed earlier about how oxidants can reach and modify these methionine residues that are structurally shielded from the medium. Since the oxidation of buried and structured methionines is known to be promoted by the previous oxidation of neighboring methionines that are more exposed or flexible [162], McKnight and colleagues have put forward the following hypothesis. Methionines 336, 337, and 339, which, according to the footprint, are more reactive than Met322 and Met323, may form part of a redox switch that is evolutionarily conserved. When the concentration of oxidants rises above a threshold, e.g., as consequence of active synapses that are mitochondria-rich, then the most accessible methionine residues may be oxidized to sulfoxide. This modification, that as we have discussed above can be structurally destabilizing, could trigger a cascade of movements leading to minor local loss of structure, but enough to eventually facilitate the access of oxidants to those methionines involved in the maintenance of the condensates.

### 5.4. Ataxin-2

Like TDP-43, ataxin-2 is an RNA-binding protein identified as a genetic determinant or risk factor for various neurological diseases, including spinocerebellar ataxia type II and amyotrophic lateral sclerosis, among others [163]. Ataxin-2 is a modular protein involved in many different cellular processes, including the regulation of RNA stability, RNA translation, regulation of cellular metabolism, and circadian rhythms [163]. Being a modular protein (Figure 3B), its size varies widely between orthologous, ranging from 301 residues in the *Paramecium tetraurelia* to 1392 amino acids in the chimp orthologous [164]. However, most orthologous proteins contain at least three well conserved domains, e.g., Lsm, LsmAD, and PAM2, and are believed to be involved in (i) mRNA binding and processing, (ii) Clathrin-mediated trans-Golgi trafficking, and (iii) interactions with poly(A)-binding protein, respectively [165]. Although a PAM2 motif has not been identified in the orthologous proteins from *Saccharomyces cerevisiae* and *C. elegans*, both proteins can interact with poly(A)-binding protein [164], most likely via LC regions found towards the C-terminal end of the protein [166]. In addition to these well characterized motifs, ataxin-2 proteins contain LC PrLDs that may play key roles in the assembly of cytoplasmic MLOs including P-bodies, SGs and neural granules [167]. In this respect, recent work from the laboratories of Benjamin Tu and Steven McKnight have provided strong evidence of the relevance of LC PrLD of Pbp1, the budding yeast orthologous of ataxin-2, in the control of LLPS in response to metabolic demands [74,128]. This work represents a breakthrough because it provided the first evidence of a fascinating new mechanism, based on the redox state of methionine residues, for regulating the assembly/disassembly of MLOs. Furthermore, this mechanism based on the reversible oxidation of methionines from the LC domain of Pbp1, provides insights into how cells couple their metabolic activity to growth conditions by remodeling their MLOs to deploy an optimal response to environmental clues. Next, we will elaborate a bit on these affirmations.

**Figure 5:**
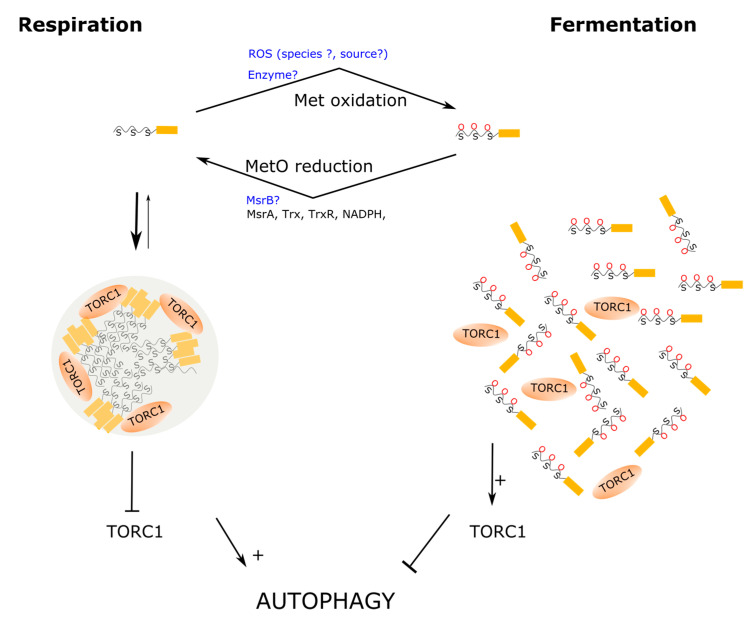
Metabolic status and autophagy level. The figure outlines the mechanism that coordinates the metabolic state (fermentation versus respiration) with the levels of autophagy (low and high, respectively). Key to this mechanism is the redox state of specific methionine residues of the Pbp1 protein. When these residues are in their reduced form, as methionines, the protein is competent to form biomolecular condensates that retain TORC1 in a non-soluble compartment. Since TORC1 has an inhibitory effect on autophagy, its sequestration within the condensates leads to the upregulation of autophagy. On the contrary, when the target methionines are oxidized to MetO, Pbp1 is unable to aggregate, and the condensates melt releasing TORC1 in the cytoplasm where it inhibits autophagy. It should be noted that within cells, methionine oxidation and MetO reduction take place through two different reactions (angled lines). That is, one reaction is not the reverse of the other, and both reactions have unrelated equilibrium constants. Labels in blue represent the aspects of the proposed mechanism that require further investigation. For instance, if ROS are the oxidants of the methionine residues, is there a particular species whose production is favored during glycolysis? Is the ROS formation compartment-dependent? Is Ppb1 oxidized in an enzyme-catalyzed manner? How relevant is, in vivo, MsrB in the reduction of oxidized Pbp1?

When nutrients are scarce and fermentable substrates are absent, yeast cells resort to autophagy. Taking advantage of this fact, Yang and coworkers used a minimal medium with lactate as a non-fermentable source of carbon to carry out a screen of yeast mutants with autophagy defects. In this way, Pbp1 was identified as key regulator of autophagy. The Pbp1-deficient mutant cells exhibited a phenotype matching that of hyperactive TORC1 signaling. TORC1 is a protein kinase that coordinates cellular metabolism with environmental cues to ensure that cells grow only under favorable conditions. Thus, when active, TORC1 stimulates anabolic pathways and inhibits catabolism through repression of autophagy [168]. To explain the mechanism through which Pbp1 inhibits TORC1, which in turn stimulates autophagy, the authors proved that Pbp1 can directly interact with endogenous Kog1, the yeast orthologous of RAPTOR, in the presence of minimal lactate medium, but not in minimal glucose medium. Since it had been reported that, under severe heat stress, Pbp1 could sequester TORC1 into SGs, preventing in this way TORC1 activation [169], Yang and coworkers addressed whether Pbp1 sequestered TORC1 into SGs in response to glucose deprivation in prototrophic cells. Although they failed to localize Pbp1 into SGs, they did observe that Pbp1 exhibited a non-uniform distribution appearing as condensates through the cell, while in glucose medium Pdb1 showed a more uniform distribution through the cytoplasm. Furthermore, treatment of the yeast cells with 1,6-hexanediol, a compound used to disrupt weak hydrophobic interactions and disturb LLPS, caused a more uniform redistribution of the Ppb1 protein, further supporting the view of this protein forming biomolecular condensates within living cells [128]. Although the results obtained using 1,6-hexanediol should be interpreted with caution because this alcohol may have potential side effects on protein kinases and protein phosphatases [170], the participation of Pbp1 into the formation of homotypic oligomerization in vivo was further proved using complementary approaches. Thus, co-immunoprecipitation experiments showed that Pbp1 was strongly self-associated in extracts from cells grown in minimum medium, in contrast to cells grown in the presence of glucose. In line with that result, fractionation experiments showed that, in cells grown with lactate, most of the Pbp1 protein was detected in the insoluble fraction, suggesting that it may form large protein assemblies under such conditions. In contrast, when cells were grown in the presence of glucose, a sizeable amount of Pbp1 was found in the soluble fraction [128]. Overall, these observations indicate that during respiratory growth Pbp1 does not localize into SGs, but it may form biomolecular condensates that may retain TORC1, upregulating, in this way, autophagy (Figure 5). Since LC PrLDs are prevalent among proteins that are prone to phase separate, the Tu and McMnight laboratories next directed their efforts towards characterizing the role of the Pbp1 LC region in self-association and LLPS.

Upon subcloning and expression of the Pbp1 LC region, the purified recombinant protein becomes phase separated sequentially into liquid-like droplets and hydrogels. In this regard, the PrLD of Ppb1 does not differ from the LC regions of many other RNA-binding proteins that undergo phase separation. The novel and exciting aspect here is that LLPS appears to be driven and controlled by a redox-dependent mechanism that relies on the abundant methionine residues present in the PrLD of Pbp1. Thus, while the aromatic amino acid tyrosine and phenylalanine have been shown to be abundant among LC domains from diverse RNA-binding proteins prone to undergo LLPS, where they play a relevant role driving the phase separation process, leading to the formation of liquid-like droplets or hydrogels [64,67,81], the LC region of Pbp1 is not enriched in aromatic amino acids. Instead, it contains an unusual abundance of methionine residues (Figure 3B). Furthermore, Kato and coworkers showed that Pbp1 methionine residues were sensitive to H_2_O_2_ oxidation both in vitro and in vivo. Thus, they reported that in vitro, oxidation of the methionine residues in the LC region to MetO led to disruption of preformed droplets, while treatment with Msrs allowed droplets to reform [73]. This redox control of condensates seems to also function in living cells as Pbp1 shifts into the soluble fraction of cellular extracts after treatment with H_2_O_2_, which inhibits autophagy, suggesting that sequestration of TORC1 by Pbp1 is controlled by the redox state (Figure 5). To further characterize the importance of these methionines, the authors generated and analyzed a series of mutants containing a variable number of substitutions of methionine by serine. Two methionine residues, Met614 and Met616, were revealed as important to the formation of fibrils in vitro. On the other hand, increasing the number of methionines substituted gradually reduced the ability of Pbp1 to induce autophagy in vivo, which correlated with an increased amount of Pbp1 in the soluble fraction of cellular extracts. Of interest, all the Met to Ser mutant combinations tested still phase-separate into droplets in vitro. However, variants containing increasing numbers of methionine substitutions significantly decreased the stability of the condensates, as the droplets melt more readily upon lowering the protein concentration [128]. Consistent with this observation, in fluorescent recovery after photobleaching experiments, the droplets formed by the Met to Ser mutants were able to recover much faster after photobleaching when compared to wild-type droplets, strongly suggesting that methionine residues in the LC domain contribute to stabilize the condensates. In particular, the segment ^591^MGFPMGGPSASPNPMMNGFAAGSMGMYMPFQPQPM^625^ within the Ppb1 PrLD, which contains eight methionine residues, including Met614 and Met616, is postulated as a key redox sensor that properly synchronizes cell physiology to the metabolic state of mitochondria [73]. Interestingly, when these eight methionine residues were changed by tyrosine instead of serine, the droplets formed in vitro were resistant to H_2_O_2_-driven melting. Furthermore, when this mutant was analyzed in the context of full-length Pbp1 within yeast cells, the Pbp1 protein fractionated preferentially into the non-soluble pellet fraction from cellular lysates, suggesting a gain of stability of the biomolecular condensates formed in vivo. Moreover, cells expressing this mutant version of the full-length Pbp1 protein also exhibited enhanced autophagy when compared to wild-type cells [73]. Overall, these findings point to an important role for methionine residues from the Ppb1 LC. On one side, methionine has a side chain well-endowed to stabilize protein–protein interactions, as we have discussed in previous sections. Consequently, the substitution of methionine by serine leads to destabilize the assemblies formed both in vitro and in vivo. On the other side, although the stabilizing function can be fulfilled by other amino acids, such as tyrosine and phenylalanine, methionine is unique in that its sulfur atom can be reversibly oxidized, providing, in this way, a suitable chemical mechanism to regulate the stability of the assemblies of which they form a part. 

The finding of a redox sensor crafted into the LC domain of Pbp1 represents a clear step forward towards the understanding of how cells synchronize their metabolic state to the growing conditions. Nevertheless, some details remain to be fully elucidated. For instance, why does glucose fermentation lead to Pbp1 methionines oxidation, while aerobic respiration allows to keep these methionines in their reduced form? Although this question has not yet been specifically addressed, in the Appendix A, we review some available data with the aim to offer some ideas that may help to guide future research.

### 5.5. Pab1

The poly(A) tail of mRNA is known to play multiple roles in the biogenesis, stability, and translation of messenger RNA. Many of these functions are mediated by the so-called poly(A)-binding protein (Pab1 in yeast). Like TDP-43 and ataxin-2, Pab1 is a modular protein containing RRMs and a methionine-rich LC (Figure 3C). However, while other RNA-binding proteins depend on LC domains or RNA for phase separation, the LC region of Pab1 is not required for demixing, and RNA inhibits it. Although the LC domain of Pab1 is not required for demixing, it plays a role in modulating (promoting) the process [14]. A defining feature of LC regions is that small and/or polar amino acids such as glycine, proline, serine, glutamine, and alanine are over-represented, while large and/or non-polar residues, such as arginine, cysteine, methionine, tryptophan, phenylalanine, valine, isoleucine, and leucine, are under-represented [171,172]. Therefore, the bias towards high frequencies of proline (19% versus a yeast-proteome average 4%) and glycine (14% versus 5%) observed in the Pab1 LC region fits the definition of LC domain. Somehow more surprising is the enrichment in methionine residues (10% versus 2%). To address the potential implications of this surprising finding, Riback and coworkers assessed the influence of several mutants on phase separation. To this end, they replaced methionyl residues either by more polar amino acids (alanine) or more hydrophobic ones (isoleucine). From these experiments, they conclude that intramolecular hydrophobic interactions drive the collapse of the LC domain, which influences the demixing temperature. This parameter represents a measure of the lower critical temperature, above which the solution separates into protein-rich and protein-depleted phases. Thus, a more hydrophobic LC domain (Met to Ile) decreases this temperature by 1.6 °C, while a more polar LC domain (Met to Ala) increases it by 2.2 [14].

As we have already discussed above, Met oxidation to MetO leads to a drastic increase in polarity. Therefore, it is straightforward to hypothesize that the redox state of the methionines found at the LC of Pab1 must exert a profound influence on the temperature triggering the formation of biocondensates. Unfortunately, since the authors of the referred work were interested in pH and temperature as stressor stimuli, they did not assay the effect of oxidant concentrations on the phase separation of this protein. Nevertheless, awaiting results from future research, a key role for methionine residues in the coordination of LLPS of this protein in response to different stimuli can be anticipated. In other words, we would like to postulate the Pab1 segment ^433^MPGQFMPPMFYGVMPPRGVPFNGPNPQQMNPMGGMPKNGM^472^ as a redox sensor, like those previously described for Pbp1 and TDP-43 [85].

## 6. Interconnection between Protein Translation, SGs, and Methionine Sulfoxidation

Cells respond to stress conditions with a global reduction of protein synthesis. Phosphorylation of the alpha subunit of the eukaryotic initiation factor 2 (eIF2) is an early event of this global response. This initiation factor is a heterotrimeric GTPase composed of three subunits (α, β and γ). This protein binds the Met-RNA_i_^Met^ to form the eIF2•GTP•Met-RNA_i_^Met^ ternary complex, which delivers Met-RNA_i_^Met^ to the small ribosomal subunit. Afterwards, the pairing between the AUG start codon and the anticodon of the Met-RNA_i_^Met^ triggers the hydrolysis of GTP by eIF2, releasing eIF2•GDP. Since eIF2•GDP cannot bind Met-RNA_i_^Met^, the complex eIF2•GDP should be converted to eIF2•GTP by the action of the exchange factor eIF2B before it can start a new initiation cycle. However, phosphorylation of eIF2α in response to stressors leads to the stabilization of the complex eIF2•GDP•eIF2B, preventing the GDP-GTP exchange. As eIF2B is limiting in cells, even a small proportion of phosphorylated eIF2α efficiently inhibits the rechange of eIF2 with GTP and stops, in this way, the translation initiation.

Stress-induced phosphorylation of eIF2α is sufficient for SG assembly [10]. The arrest of translation initiation is accompanied by polysome disassembly, which leads to an increased concentration of uncoated mRNAs in the cytoplasm. In this way, uncoated mRNAs are available to bind to RNA-binding proteins such as G3BP1, a scaffold protein that functions as a tunable switch that triggers LLPS to assemble SGs in response to a rise in intracellular free mRNA concentrations [13]. Of interest in the context of the current review, Met222 from eIF2α has been reported to suffer extensive oxidation to MetO after treating cells with H_2_O_2_ [173]. In a previous proteome-wide study, we addressed the potential for crosstalk between serine phosphorylation and methionine sulfoxidation. In that study, we showed that the oxidation of methionines harbored within phosphorylation motifs in response to oxidative stress is a process highly selective among stress-related proteins, including eIF2α, ataxin-2, and other proteins belonging to SGs [141]. Subsequent evolutionary analyses involving over 200 eukaryotic species suggested a tight relationship between sulfoxidation and phosphorylation among these SGs components. Thus, methionine and serine residues known to undergo posttranslational modification were shown to exhibit strong coevolutionary links [143]. Herein, molecular coevolution between two positions in a protein should be understood as the ability for amino acid substitution at one of these positions to affect the rates of substitution at the other position [174]. Overall, these studies point to a relationship between methionine oxidation and serine phosphorylation in proteins from SGs. However, the molecular mechanisms relating these two PTMs and the functional impact of this crosstalk on the regulation of SGs assembly/disassembly remain to be elucidated.

During stress, cells downregulate the bulk rate of protein synthesis [175]. However, transcripts bearing elements that evade eIF2α, e.g., possessing internal ribosome entry sites [176], are selectively translated under these stress conditions, as they produce proteins that help cells to cope with the stressful conditions. Thus, while SGs are enriched in translational arrested mRNAs released from polysomes in response to the stress stimulus, a subset of mRNAs remains actively translated and excluded from SGs. Sulfoxidation of the methionines present in the proteins encoded by this subset of transcripts seems to be an important part of the adaptive response deployed by cells to protect themselves from stress-induced damage. In this respect, a number of reports from diverse laboratories has established the view that tRNA misacylation with methionine is actively up-regulated in response to oxidative stress [117,177,178]. Thus, upon oxidative stress, methionyl-tRNA synthetase can catalyze the charge of non-cognate tRNA with methionine. Furthermore, the misacylated tRNA are used in translation replacing, in this way, non-methionine residues in proteins at strategic locations. There exists a body of evidence supporting the view of methionines exposed on the protein surface as endogenous antioxidants [115,179]. Therefore, stress-induced mismethionylation of non-methionyl tRNAs can enhance the known protective function of genetically encoded methionine residues against oxidants. The biological relevance of adaptive misacylation is reflected in the fact that this phenomenon is conserved in prokaryotes [180], fungi [178], and mammals [116].

## 7. Concluding Remarks

MLOs are non-stoichiometric supramolecular assemblies that contribute to cellular compartmentalization in a dynamic way. These biomolecular condensates that can be quickly assembled and disassembled in response to diverse stimuli are involved in many and diverse cellular processes. Although some of these MLOs were described more than a century ago, until recently, it has not been shown that LLPS, driven by multivalent interactions between scaffold proteins, is a fundamental organizing principle for MLOs.

Taking advantage of this physicochemical framework, laboratories around the world have been addressing how cells regulate the assembly/disassembly of these structures, the molecular composition and physical properties of the condensates, and the role played by these supramolecular structures in the cellular physiology. In the past several years, many high-impact works have been published that together allow us to draw some general principles. First, the molecular interactions stabilizing the biomolecular condensates (quinary structure), and therefore driving LLPS, are the same ones that stabilize the tertiary and quaternary structures of proteins. Hence, hydrogen bonds, salt bridges, hydrophobic effect, cation-π, π−π, domain–domain, and domain–linear motif interactions have all been described to make important contributions to the phase separation process. Not surprisingly, the ability to undergo LLPS may be a universal property of proteins provided the right conditions. However, only a selected group of proteins, referred to as scaffold proteins, have been evolutionary selected to trigger and organize the formation of a quinary structure in a regulated and biologically meaningful manner. Although the relative contribution of the different types of molecular interactions to the formation of condensates can vary widely from one protein to another, scaffold proteins share some features that are relevant for the phase separation process. Thus, many of them are RNA-binding proteins with a domain architecture. Beside one or several RRMs, these proteins often contain IDRs of low complexity that together endow the protein with a multivalent binding capacity. Indeed, the residues located in these LC regions seem to play key roles in establishing residue–residue interactions, being able to modulate the demixing process via competition between intra- and intermolecular interactions. Since a biased amino acid composition is a hallmark of LC domains, the amino acid composition of these regions can determine the type and strength of the interactions that can be stablished. In addition, many of these residues represent a target of diverse PTMs that, in a coordinated fashion, regulate the aggregation ability of the whole protein.

Although in most LC domains methionine is not a prominent amino acid, there exists a group of scaffold proteins that present LC regions with exceptionally high abundance of methionine residues. This is the case at least for TDP43, ataxin-2, and Pab1. Methionine residues can be reversibly oxidized to MetO. This modification, which drastically changes the physicochemical properties of the side chain, can be exploited to locate a redox sensor within the LC region of these proteins. In line with this view, preformed droplets of TDP43 or ataxin-2 can be melted by the oxidation of their methionines with hydrogen peroxide. Conversely, the reduction of MetO back to methionine restores the capacity of these proteins to form condensates via LLPS. The realization that LLPS can be controlled by reversible methionine oxidation, and that the methionine-rich LC domains of these proteins provide a device able to sense and respond to cellular stimuli, open fascinating possibilities for the methionine-based control of MLOs function. What we have learned from the experimental characterization of proteins, such as ataxin-2, TDP-43, and Pab1, should guide and encourage the search for similar redox sensors in other scaffold proteins.

## Appendix A

In the main text of the current review, we have seen that a redox sensor crafted into the LC domain of Pbp1 allows cells to coordinate their metabolic state with the growing conditions. However, there are important details about the proposed mechanisms that remain to be elucidated. This appendix revolves around the question: Why does glucose fermentation lead to Pbp1 methionines oxidation while aerobic respiration allows to keep these methionines in their reduced form? Since no experiments have been described that specifically address this issue, the most we can offer at this point is to jot down some ideas so that they can guide future research.

It has been observed that respiratory chain deficiencies in mammals induce mTORC1 signaling [181]. On the other hand, yeast cells exposed to exogenous H_2_O_2_ or treated with antimycin A (an inhibitor of the cytochrome bc1 complex) or cyanide (an inhibitor of the cytochrome c oxidase complex) each lead to the inhibition of autophagy [73,182]. In all these situations, reactive oxygen species (ROS) levels are known to be higher than those found in cells growing under physiological conditions. Regardless, they resort to aerobic fermentation or respiration. Thus, under conditions of oxidative stress, we expect Pbp1 methionine residues to be oxidized to MetO, which would result in the disassembly of Pbp1 condensates and the release of the sequestered TORC1 that can now exert is inhibitory effect on autophagy. Thus, what we have learned about Pbp1 and its ability to undergo phase separation in a redox-regulated way allows us to explain what we observe under oxidative stress conditions. However, the problem we face now is to explain why this set of methionines that constitutes the redox sensor of Pbp1 remains reduced during aerobic respiration while it becomes oxidized when the metabolic state changes to aerobic fermentation.

It is now well established that ROS, when produced in a spatiotemporally controlled form, can function as signaling molecules [183]. An obvious question to ask is whether cellular ROS levels are higher in cells grown in glucose than in cells grown in non-fermentable carbon sources. Although this possibility cannot be completely ruled out, it seems unlikely. Thus, Cabiscol and coworkers found that the levels of protein carbonyl content, an indicator of oxidative damage, in crude extracts of yeast cells grown in YPG (glycerol as carbon source) were 1.6 times higher than those in cells grown in YPD (glucose as carbon source) [184]. Nevertheless, we must bear in mind that different methods of estimating ROS levels can yield different results. In this respect, Maslanka et al. reported that, when the ROS levels were assessed using the probe dihydroethidium, which is oxidized by superoxide anion to fluorescent ethidium, the yeast cultivated in glycerol medium showed lower ROS levels than those cells grown in glucose [185]. However, when these same authors used another probe, H_2_DCF-DA, which is not substrate-specific, to evaluate the ROS content, then the highest level of ROS was observed in cells grown in glycerol.

This mixture of somewhat conflicting results illustrates the complexity of finding the physiological Pbp1 oxidant. In addition, spatial compartmentalization of ROS production adds a further layer of complexity to our problem [186,187]. In other words, to conveniently oxidize Ppb1 methionyl residues in response to metabolic changes, it may be critical to produce ROS in the right compartment rather than increase total cellular ROS levels. Interestingly, when yeast mitochondria are incubated with either succinate or malate, low levels of hydrogen peroxide are produced. However, if exogenous NADH is added, larger amounts of H_2_O_2_ are produced at the external NADH dehydrogenases [188]. Unlike mammalian mitochondria, yeast mitochondria oxidize cytosolic NADH directly via two external NADH dehydrogenase, Nde1 and Nde2, which are located at the inner mitochondrial membrane with their active sites facing the intermembrane space [189]. Since the mitochondrial inner membrane is impermeable to NADH, they have been proposed to oxidize cytosolic NADH produced via glycolysis [190]. On the other hand, yeast cells can also produce H_2_O_2_ via a NADPH oxidase-like enzyme named Yno1p. Thus, it has been shown that Yno1p-derived H_2_O_2_ regulates processes controlled by MAPK pathways [191]. In summary, the oxidation of the Pbp1 LC region by a specific oxidant produced in a controlled way is a possibility that remains to be explored.

An alternative, although highly speculative as there is currently no direct evidence to support it, is that methionine residues from the Pbp1 redox sensor may be oxidized in a highly specific manner through an enzyme-catalyzed reaction. Indeed, flavin-containing monooxygenases (FMOs) have been shown to oxidize protein-bound methionines using molecular oxygen and NADPH as co-substrates [192]. In animals, MICALs, a family of FMO enzymes [193], have been characterized as NADPH-dependent methionine sulfoxidase exhibiting a high substrate specificity, and as being involved in the control of F-acting assembling/disassembling [121,122,123] as well as in the regulation of CaMKII [125]. In the fungus *Aspergillus nidulans*, a FMO termed FmoB has been identified as responsible for the inactivation of the transcription factor NirA via oxidation of a single methionine residue in its nuclear export signal [120]. Therefore, although the currently characterized FMO from *S. cerevisiae* can oxidize thiol-compounds but not methionine [194], the existence of enzyme-catalyzed methionine oxidations in yeast cannot be ruled out.

Hitherto, we have focused our attention on the oxidation of the methionines forming part of the redox sensor from the Pbp1 PrLD. However, it is important to realize that what is relevant for the homotypic aggregation of Pbp1 is the stationary redox status of these methionines, which is the result of the balance between two different reactions: methionine oxidation, which has been discussed above, and MetO reduction, which will be considered next.

Methionine sulfoxide has an asymmetric sulfur center. Thus, when protein-bound methionine residues are oxidized by ROS, generally a racemate of two diasteroisomers, methionine-*S*-sulfoxide (Met-S-SO) and methionine-*R*-sulfoxide (Met-R-SO), is formed. However, different oxidants (different oxygen reactive species) may exhibit different grades of stereospecificity [195]. In any case, these two stereoisomers can be reduced back to methionine by two evolutionarily unrelated stereospecific enzymes designated as MsrA and MsrB, respectively [142,196]. The budding yeast *S. cerevisiae* has single MsrA and MsrB genes. Thus, in this organism, MsrA is the main enzyme that reduces Met-S-SO residues and its primarily detected in the cytoplasm, while MsrB is responsible for reducing Met-R-SO and is mostly found in mitochondria [197]. Interestingly, MsrA, despite mainly being a cytosolic enzyme, has been proven essential to maintain functional mitochondria. Thus, deletion mutants lacking this isoform show the same levels of mitochondria, but these mitochondria were not respiration-competent as judged by a low accumulation of the MitoRed marker (17% with respect to wild-type), which selectively accumulates within mitochondria able to maintain their membrane potential [197]. This result is coherent with the working hypothesis that the lack of MsrA activity in the cytoplasm of these mutant cells prevents the reduction of MetO from Pbp1, which, in turn, should block the condensation of this protein when the cell shifts towards a respiration-dependent metabolic state. If this is the case, an easy to test prediction is that these mutants must exhibit hyperactive TORC1 signaling, including depressed autophagy, even during respiratory growth, which would explain the fact that the number of mitochondria is maintained in the mutants, while these mitochondria have lost their functionality.
